# What the Overpotential Does Not Tell You: Diagnosing Non-Comparability Across Hydrogen and Oxygen Evolution Benchmarking Metrics

**DOI:** 10.3390/molecules31203696

**Published:** 2026-10-15

**Authors:** Raymond Tichaona Taziwa, Jude Nodebechukwunso Ike

**Affiliations:** Department of Applied Sciences, Faculty of Natural Sciences, Walter Sisulu University, Buffalo City Campus, East London 5200, South Africa

**Keywords:** oxygen evolution reaction, hydrogen evolution reaction, electrocatalyst benchmarking, reproducibility, Tafel slope, turnover frequency

## Abstract

Overpotential at 10 mA cm^−2^ (η_10_) is the default benchmark for hydrogen evolution reaction (HER) and oxygen evolution reaction (OER) electrocatalysts, but the number is rarely comparable across studies. This Perspective identifies six measurement and reporting stages, from synthesis reproducibility to the measurement platform itself, at which undisclosed methodological choices shift reported activity by tens to hundreds of millivolts without changing the catalyst. The benchmark’s device-context origin matches neither laboratory practice nor industrial current density, and its geometric normalization is sensitive to catalyst loading. Comparable artefacts recur in Tafel slope extraction, electrochemically active surface area, turnover frequency, and Faradaic efficiency, each traced to a specific undisclosed choice and a minimum reporting requirement. We test the framework on literature values checked against their primary sources. Of 11 NiFe oxyhydroxide reports, five give η_10_ on a matched basis (1 M KOH, room temperature), and these span 134 to 372 mV. For commercial Pt/C, the field’s own reference material, five matched-basis reports span 33 to 93 mV, and loading is stated directly in only two of the seven reports checked. We close with a concise reporting checklist organized by metric.

## 1. Introduction

Water electrolysis is regaining ground as the most direct route to green hydrogen. Recent reviews have traced its path from textbook electrochemistry to industrial deployment in detail [1]. Materials work continues on both fronts, from engineering the dynamic reactant–electrode interface [2] to doping NiFe layered double hydroxides for faster alkaline water splitting [3]. But progress on the materials side rests on a fragile foundation. The overpotential values used to rank hydrogen evolution reaction (HER) and oxygen evolution reaction (OER) catalysts are rarely comparable across studies, even when the catalysts themselves are nominally identical. A 2025 global interlaboratory study makes the point with numbers. Ten groups worldwide, each with at least 3 years of post-master’s experience in electrocatalysis, received three literature-derived synthesis procedures for a nickel-iron OER catalyst. They worked blind to the expected performance. No communication between groups was allowed [4]. The phases matched by X-ray diffraction (XRD). Every electrode was OER active. And yet the current density and O2 production rate varied by roughly two orders of magnitude across all three procedures, with an interlaboratory standard deviation about three times larger than the intralaboratory one [4]. When the spread between “the same” material made by different hands rivals the spread reported between genuinely different materials, the overpotential’s usefulness for ranking catalysts is undermined. Much of this spread has a thermodynamic origin that gets waved off as noise. Overpotential is conventionally referenced to the reversible hydrogen electrode through the Nernst equation, E_TD = E° − (RT/zF) ln Q, assuming a fixed equilibrium potential of 1.229 V and a nominal electrolyte pH of 14 at 25 °C [5]. In practice, a survey of the electrocatalysis literature found reported pH values for 1 M KOH ranging from 13.5 to 14 at the same nominal temperature, a roughly threefold difference in OH^−^ activity that most papers neither measure nor state [5]. For a representative HER catalyst, calculating overpotential with pH 13.5 instead of 14 changes the result from 50 mV to 20 mV, a 60% swing that alone moves the apparent efficiency loss from about 4% down to under 2% [5]. Much of this ambiguity traces back to ordinary temperature drift. A 5 °C difference shifts the pH of concentrated KOH by about 0.17 units, and uncontrolled room temperature measurements scatter across a 13.7 to 14.1 range before any current has even been drawn [5].

Propagating a measured pH of 13.83 instead of the assumed 14 shifts the OER equilibrium potential from 1.229 V down to 1.208 V vs. the reversible hydrogen electrode (RHE). Neglecting the coupled temperature-pH correction introduces a systematic overpotential error of roughly 7 to 10 mV at minimum, growing to 10 to 20 mV as deviations widen, on top of a further 5 to 15 mV from uncalibrated, temperature-independent reference electrode conversions [6]. The problem does not stop at the bulk electrolyte. A diffusion-reaction model of the OER catalyst-electrolyte interface shows local pH tracks bulk pH only below about 10 mA cm^−2^; above that threshold, proton production outpaces hydroxide or buffer transport, and the model predicts that at industrially relevant current densities near 1 A cm^−2^ the local environment turns acidic, approaching pH 1, regardless of how basic the bulk solution is [7]. Two electrodes running the identical bulk electrolyte at different current densities are, by this measure, not operating at the same effective pH at all. Both the temperature pH and the interfacial pH work point to the same gap: the field has no standard way to catch either error before publication [5,6].

The same interlaboratory study borrowed a pharmaceutical industry design of experiments approach and applied it to a widely used NiFe electrodeposition procedure, varying six parameters (iron nitrate ratio and concentration, deposition time, convection, polishing, and post-deposition solution time) across 76 catalyst syntheses and test runs performed by one experimentalist in one laboratory [4]. For the first voltammetric cycle, deposition time and solution time dominated, each with a probability above 99.9% of being real. By the tenth cycle, polishing had taken over as the most influential parameter, traced to film detachment on smooth electrodes that begins in cycle one and quietly skews the statistics from there on. Several two-way interaction terms involving convection were significant too, even though convection’s main effect was modest, an effect a one parameter at a time analysis would have missed entirely [4]. This magnitude of variation came from undisclosed parameters inside a single well-controlled lab, using a procedure that has itself been proposed as an internal benchmark. Non-reproducibility in electrocatalysis is not just a matter of sloppy technique. Even with synthesis and testing held fixed, the way a measured curve gets turned into a reported number carries its own hidden variability. A systematic critical assessment of the field’s standard activity parameters found that the percentage of ohmic (iR) drop compensation applied to a polarization curve, a quantity often left unstated, has an outsized effect on the resulting overpotential. For a cobalt phosphide OER catalyst, compensated and uncompensated overpotentials differed by only about 13 mV at 10 mA cm^−2^ but by more than 150 mV at 100 mA cm^−2^. For a RuO_2_ electrode, the reported overpotential at 25 mA cm^−2^ slid continuously from 437 mV uncompensated down to 382 mV at full compensation, depending on which fraction the author chose [8]. The method used to apply that compensation is itself a hidden variable. Manually calculated 90% iR compensation and the electrochemical workstation’s automated 90% compensation, run on the same NiFe layered double hydroxide (LDH) electrode under identical conditions, landed 47 mV apart [8]. A metrology-focused perspective on electrochemical measurement makes the underlying point explicit: whether iR compensation should even be applied depends on what is actually being measured. Correcting it out is appropriate when the uncompensated resistance is a method artifact, and inappropriate, “only serves to obfuscate,” when that resistance is intrinsic to the measurand itself, such as the real operating voltage of an electrolyzer cell [9]. Tafel slopes carry the same problem. The same RuO_2_ electrode gave markedly different slopes depending on whether the voltammogram was recorded at 5 or 100 mV/s, and for a Pt/C catalyst, slopes derived from linear sweep voltammetry (LSV), chronoamperometry, and impedance spectroscopy were 42, 33, and 28 mV/dec, respectively. Only the impedance-derived value landed close to the theoretical 30 mV/dec [8]. Turnover frequency (TOF) carries the largest uncertainty of all. Calculating it from the full mass of loaded catalyst instead of a redox peak-derived count of actually active sites can shift the reported value by two to three orders of magnitude, and the closely related electrochemically active surface area (ECSA) is, more generally, “often very challenging to measure in a reproducible manner” even before it is used to normalize anything else [8,9]. None of this is a reporting error in the usual sense: each method is legitimate on its own, but the choice among them is rarely disclosed, and that choice can dominate the catalyst-to-catalyst differences the field treats as meaningful. Faradaic efficiency carries the same problem in a different guise: when a value is reported at all, the method behind it, gas chromatography, water displacement, or rotating ring-disk collection, is frequently left unstated, despite the three carrying materially different error profiles (Section 7). Counting active sites in the first place adds another, independent layer of disagreement. A recent kinetic probe study of iron single-atom Fe-N-C oxygen reduction catalysts benchmarked a hydroquinone oxidation method against three independently established site-counting techniques, CO pulse chemisorption, electrochemical stripping of NO derived from nitrite, and ^57^Fe Mössbauer spectroscopy, across catalysts made by different synthetic routes [10]. Even the two most concordant methods, hydroquinone oxidation and CO chemisorption, disagreed with a mean absolute error (MAE) of 1.4 × 10^−5^ mol g^−1^ and a mean percentage error (MPE) of 26%. Agreement with NO stripping was worse still (MAE 2.3 × 10^−5^ mol g^−1^, MPE 53%), and Mössbauer spectroscopy showed comparable disagreement plus systematic outliers whenever FeNx sites were a minority species [10]. These methods were applied to the same catalysts, although they do not all probe the same subset of sites, and they still disagree by tens of percent on how many sites are doing the chemistry. Every reported turnover frequency, and every activity normalized against it, inherits whichever of these disagreements the author happened to pick. Watching the catalyst under working conditions does not fix any of this. If anything, it adds to the pile. A 2026 perspective, built from a U.S. National Science Foundation (NSF)-convened workshop on rigor and reproducibility in operando electrocatalysis, lays out recurring, platform-agnostic sources of error across X-ray absorption spectroscopy (XAS), vibrational spectroscopy (surface-enhanced infrared absorption spectroscopy, SEIRAS; surface-enhanced Raman spectroscopy, SERS; shell-isolated nanoparticle-enhanced Raman spectroscopy, SHINERS; sum-frequency generation, SFG), electrochemical mass spectrometry, and liquid-cell electron microscopy [11]. These include mass transport limitations built into thin-layer operando cells; non-uniform current distribution, governed by the ratio of charge transfer to ohmic resistance (the Wagner number); orders-of-magnitude mismatches between the millisecond timescale of the electrochemical event and the acquisition time of the spectroscopic or imaging probe observing it; reference and counter electrode artifacts that drift by more than 100 mV over the course of an experiment; and probe-induced beam damage that can itself drive the redox chemistry under study [11]. The perspective’s central recommendation, transparent and technique-specific reporting rather than forced standardization of cell geometry, makes a point worth carrying forward here: these are measurement artifacts stacked on top of the synthesis, processing, and site-counting problems above, not separate from them.

And underneath all of it, the catalyst itself is not guaranteed to sit still. Rigorously iron-free NiOOH shows essentially no OER activity below 400 mV overpotential. Intentional or incidental iron incorporation, often carried in from unpurified KOH, boosts activity by more than an order of magnitude without a proportional change in conductivity [12]. A catalyst screened before and after such reconstruction is not, electrochemically, the same material. Direct operando evidence bears this out. Time-resolved diffraction and pair distribution function analysis of a NiFe layered double hydroxide catalyst under OER conditions show that the crystallite radius collapses from roughly 34 Å to 16 Å within the first potential step into the active regime, and that the reduction never recovers when the potential is stepped back down [13]. It gets reported as one anyway. None of this means these comparisons are beyond rescue. It means the correction a given comparison needs depends on where in the measurement pipeline that comparison actually sits, and the field currently treats every correction as equally optional, regardless of stage. We organize the artifacts documented above into six pipeline stages, from synthesis through the measurement platform itself, and map each stage against the type of claim it threatens (Table 1). A same-lab ranking claim lives or dies on stages 3 and 4, where iR compensation and normalization basis are chosen rather than reported. A cross-literature record claim inherits stage 1 in full; no downstream correction repairs a two-order-of-magnitude spread in as-synthesized reproducibility. A mechanistic claim built on turnover frequency inherits whichever site-counting disagreement its author happened to pick at stage 4. A high current density or industrial relevance claim is exposed to stages 2 and 6, both negligible at the densities most catalysts are actually screened at, and both large at the densities relevance is claimed for. This is not another call for more disclosure. It is a claim about which disclosure a given comparison specifically needs, a distinction that the field’s existing checklists do not make. Papers that advance more than one claim at once inherit more than one set of vulnerabilities, and Table 1 is built to be read stage by stage rather than as a single verdict on a study as a whole. Individual elements of this pipeline are not unrecognized. McCrory et al. established the η_10_ convention itself and, within the same protocol, the ±1-order-of-magnitude ECSA uncertainty this Perspective revisits in Section 5 [14]. Voiry et al. and Wang et al. each proposed reporting checklists for electrocatalytic performance [15,16]. Anantharaj and Noda set out practical dos and don’ts for screening, including steady-state Tafel analysis and site-based normalization of TOF [17]. Kempler and Nielander set thermodynamic criteria for when Faradaic efficiency measurement is mandatory rather than optional [18]. Tackett et al. catalogued platform-agnostic artefacts across four operando techniques [11]. What none of these treatments does is connect the six stages to each other or to the specific claim a given comparison is trying to support. Each existing checklist is additive: a list of items to disclose for one metric, applied uniformly regardless of what the paper is claiming. None distinguishes a same-lab ranking claim, which lives or dies on the data-processing and normalization choices at stages 3–4, from a cross-literature record claim, which no downstream correction can rescue once stage-1 reproducibility is compromised. The framework introduced here is not another checklist alongside those above; it is the missing cross-reference between them, tracing each artefact to the stage of the measurement pipeline at which it originates and reading that stage against claim type (Table 1). That cross-reference, not any additional disclosure item, is the gap this perspective addresses.

We test this framework against real numbers, not just against the artefact reports above. Section 8 builds two literature sets under one rule: every value comes from the primary source, none from an abstract or a review’s summary table. Of 11 NiFe oxyhydroxide reports checked, five give η_10_ in 1 M KOH at room temperature, and these five still span 134 to 372 mV. Commercial Pt/C, checked across seven independent sources, gives five matched-basis values spanning 33 to 93 mV, with Tafel slopes from 30 to 105 mV dec^−1^ and loading stated directly in only two of the seven. A reference material that moves this much between laboratories is direct evidence that the checklist we close with addresses a real gap.

## 2. Methodology

This is a literature-based critical analysis, not a primary experimental study. The methodology below covers how sources were selected and how the case study in Section 8 was built, rather than materials or instrumentation.

**Scope.** This Perspective draws on primary experimental and methodological literature on HER/OER benchmarking published through January 2026, spanning major journals in electrochemistry, materials, and energy, as well as directly relevant patents. Sources were included where they report a quantified, reproducible magnitude for a specific artifact, a millivolt shift, an order-of-magnitude discrepancy, or a percentage error, backed by original measurements rather than a value taken from elsewhere. For the reconstruction and site-counting evidence in particular, sources span several catalyst classes: oxide, hydroxide, phosphide, sulfide, metal–organic framework, so the claim is not built on one material alone.

**The case study**. Section 8 applies the framework below to two sets of real published data: reports of plain NiFe oxyhydroxide catalysts for OER, and reports of commercial Pt/C for HER, both under matched alkaline conditions. Every value used there comes directly from the source paper, not from an abstract, a review’s summary table, or a citation chain. Where a source’s own reported number could not be confirmed, it is named but excluded from the totals, a distinction kept visible throughout Section 8.

**Figure 1:**
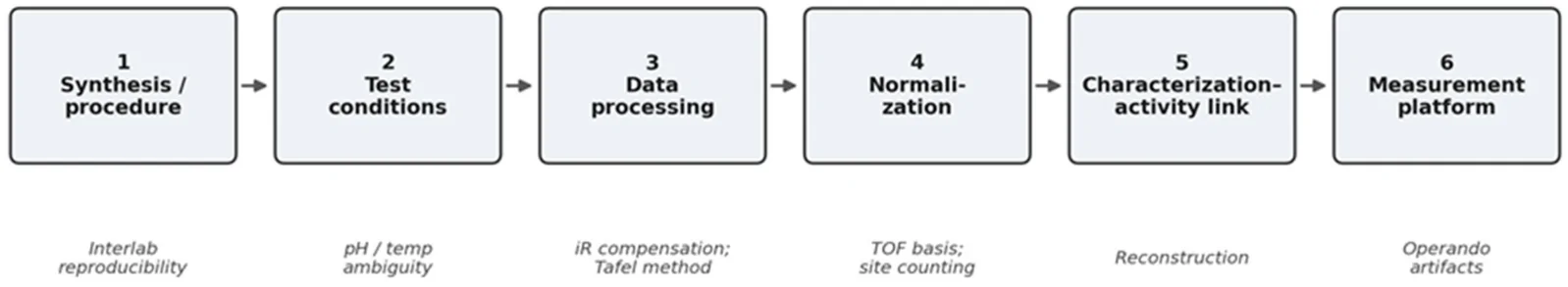
The six-stage measurement pipeline matches the stages in Table 1.

Analytical framework. The six-stage pipeline (Table 1, Figure 1) follows the order in which an experimenter makes choices: how the catalyst is made (stage 1), the conditions under which it is tested (2), how raw data become a number (3), what that number is normalized by (4), how the characterized material relates to the active one (5), and which platform observes it (6). The stages are defined by position in that workflow, not derived statistically from the artefact literature. Each was then populated with quantified primary or methodological reports. Stages 2 to 5 each rest on at least two independent sources. Stage 1 rests chiefly on one interlaboratory study [4], and stage 6 on one multi-group perspective [11] that synthesizes many primary reports. We retain both because they mark distinct points in the workflow, and Table 1 notes their narrower evidence base. A coarser or finer division is possible; we found six to be the smallest that keeps choices made at different points, by different people or instruments, in separate rows. The stages are organized around the class of comparative claim, same-lab ranking, cross-literature record, mechanistic, industrial-relevance, before/after stability, or operando/working-condition, which stage most directly threatens. Section 3, Section 4, Section 5, Section 6 and Section 7 each address one metric (overpotential, Tafel slope, ECSA, turnover frequency, Faradaic efficiency) but, where applicable, organize their internal discussion around the same six stages, so a reader assessing a specific published comparison can identify which stage is load-bearing for that comparison’s validity.

## 3. Overpotential and the 10 mA cm^−2^ Benchmarking Convention

### 3.1. Definition and Thermodynamic Context

The overpotential η at the cathode (HER) or anode (OER) is defined as the difference between the electrode potential applied under current flow and the thermodynamic equilibrium potential for the half-reaction at the operating pH and temperature.: Mathematically, it can be written as:

η = *E*_applied_ − *E*_eq_(pH, T) (1)

where potentials are expressed on the RHE scale, which corrects the standard hydrogen electrode potential for pH and temperature as E(RHE) = E(SHE) + (RT/F)ln(10) × pH, numerically 0.0592 × pH at 25 °C but rising to 0.0701 × pH at 80 °C under industrially relevant alkaline water electrolysis (AWE) conditions. On the RHE scale, the HER equilibrium potential is 0.00 V, and the OER equilibrium potential is +1.23 V at all pH values at 25 °C, making cross-comparison of catalyst activity in acid and alkaline electrolytes formally valid under standard conditions. Temperature also moves the thermodynamic reference. On the RHE scale, the HER equilibrium potential remains 0 V by definition, but the OER equilibrium potential falls by about 0.85 mV K^−1^, from 1.23 V at 25 °C to roughly 1.18 V at 80 °C [1]. An OER overpotential at 80 °C calculated against 1.23 V is therefore overstated by about 45 to 50 mV. A second error enters when potentials measured against a conventional reference electrode such as Hg/HgO are converted to RHE with room-temperature constants. Its size depends on the electrode’s own temperature coefficient, and it is avoided by calibrating against a hydrogen electrode in the working electrolyte at the operating temperature [6].

### 3.2. The 10 mA cm^−2^ Benchmark: Origin, Scope, and Limitations

The selection of 10 mA cm^−2^ as the canonical current density at which overpotential is reported was established by McCrory, Jung, Peters, and Jaramillo in their 2013 benchmarking study [14]. The threshold was chosen because it corresponds to the geometric anodic current density expected at the working electrode of a 10%-efficient integrated solar-to-fuels device operating under one-sun illumination, a device-context rationale that distinguished it from an arbitrary convenience value. Under the standardized conditions of the McCrory protocol (glassy carbon substrate, 1 M NaOH or 1 M H_2_SO_4_, rotating disk electrode (RDE) voltammetry at 1600 rpm and 10 mV s^−1^), the application of this benchmark to ten electrodeposited earth-abundant OER catalysts in alkaline media revealed that all achieved 10 mA cm^−2^ within an 80 mV overpotential window (0.35–0.43 V), a result consistent with the interpretation that much of the activity spread visible in unstandardized literature reports arises from protocol differences rather than genuine differences in intrinsic catalyst activity [14]. Two limitations of the η_10_ benchmark are fundamental and cannot be resolved by improved experimental techniques. First, 10 mA cm^−2^ is calibrated to a device operating at 1–2% of the current density at which commercial alkaline and proton exchange membrane (PEM) electrolyzers actually operate (200–2000 mA cm^−2^). A catalyst that achieves η_10_ = 300 mV but collapses to unacceptable voltage at 500 mA cm^−2^ provides no electrolyzer value; conversely, a catalyst with η_10_ = 350 mV but a shallower Tafel slope may outperform the first catalyst at all industrially relevant current densities. The appropriate benchmarking metric for industrial translation is η at ≥500 mA cm^−2^ in the electrolyte at a temperature matching the target application [15,16]. Second, under geometric normalization (the most common reporting basis), η_10_ is strongly sensitive to catalyst loading: for a catalyst with a Tafel slope of 40–60 mV dec^−1^, a tenfold increase in active loading lowers the apparent η_10_ by one Tafel slope, 40 to 60 mV, sufficient to transform an apparently mediocre catalyst into a record-breaking one without any change in intrinsic activity [17,19]. Direct evidence confirms this is not a hypothetical concern. On electrodeposited Ni_80_Fe_20_OOH, catalyst layers of different loading show systematically divergent Tafel slopes at the same overpotential, and literature-tabulated Tafel slopes for NiFe layered double hydroxides of varying loading span 27–71 mV dec^−1^ in similar electrolytes [20]. On an independent catalyst system, Co_3_O_4_, increasing electrode loading from 128 to 510 μg cm^−2^ nearly halved the measured turnover frequency, because increased stacking lengthens the electron-transport path and leaves interior catalyst poorly wetted by electrolyte even as nominal loading rises [21]. The combination of these two limitations produces a benchmarking convention that simultaneously overstates intrinsic activity differences (through loading sensitivity) and understates industrial relevance (through low current density calibration). Wei and Xu provide a comprehensive quantitative analysis of the conditions under which η_10_ is meaningful, specifically when measurements are performed at ECSA-normalized rather than geometric current density, at matched and disclosed loadings, and in electrolytes matching the target application [19]. Absent these conditions, η_10_ comparisons between laboratories are not interpretable as comparisons of catalyst activity.

### 3.3. The Loading Artefact and Three-Dimensional Substrate Normalization

For three-dimensional, high-surface-area electrode architectures (nickel foam, carbon cloth, nickel mesh, and all self-supported electrode configurations), the macroscopic electrode’s geometric area is a poor proxy for the electrochemically accessible area. Reporting η_10_ at geometric current density for such electrodes systematically underestimates the true current per unit real surface area, inflating apparent performance relative to equivalent planar catalysts at lower loading. McCrory et al. chose smooth glassy carbon as their benchmarking substrate for its relative electrochemical inertness at moderate OER overpotentials, not explicitly to control for the loading artefact [14]; the choice nonetheless incidentally avoided it. In contrast, the widespread adoption of η_10_ as a universal benchmark while simultaneously shifting to three-dimensional substrates has effectively abandoned this control. A meaningful comparison of η_10_ values across laboratories therefore requires catalyst loading to be matched and reported alongside the performance metric [19]. In the absence of loading disclosure, any η_10_ comparison is uninterpretable. Table 2 makes this concrete across seven representative NiFe OER reports. Substrate ranges from smooth glassy carbon and gold to rough nickel foam and stainless steel, geometries with very different real-to-geometric surface area ratios, while electrolyte concentration spans a tenfold range. Loading is disclosed as a mass per area in only one of the seven (Dionigi et al.); Louie and Bell give a film thickness, and Geerts et al. a nominal monolayer coverage as rough substitutes, and four give nothing at all. Three of the seven (Dionigi et al., Wang et al., and Tang et al.) report no Tafel slope either, despite all three being LSV-based OER studies, so the reader cannot even check kinetics as a cross-check on the reported η_10_. One source (Chen et al.) shows the same headline-number problem documented for Jeon et al. in Section 8.1: their own abstract leads with 182 mV at 50 mA cm^−2^, while the number most other papers cite from this work, 134 mV, appears only in the results text at 10 mA cm^−2^.

## 4. Tafel Slope Analysis and Potentiodynamic Extraction Artefacts

### 4.1. Theoretical Basis and OER-Specific Considerations

The Tafel slope *b* = dη/d(log j) is the fundamental kinetic diagnostic for electrocatalytic reactions. For the HER, textbook limiting values are 120 mV dec^−1^ (Volmer step rate-determining, low H* coverage), 40 mV dec^−1^ (Heyrovsky rate-determining step (RDS), low coverage), and 30 mV dec^−1^ (Tafel recombination step RDS). The Tafel equation appears as:

*j* = *j*_0_ exp(α_a_*F*η/*RT*) (2)

which represents the large-overpotential limit of the Butler–Volmer expression, valid when the reverse reaction rate is negligible. The effective Tafel slope is known to transition between textbook limiting values over narrow overpotential windows, and a measured slope of 120 mV dec^−1^ is mechanistically ambiguous, consistent with either the Volmer RDS at any coverage or the Heyrovsky RDS at high coverage [22]. For the OER, the four-electron adsorbate evolution mechanism (AEM) sequence does not yield a single set of universally agreed Tafel slope assignments. In practice, NiFe-OOH and related alkaline OER catalysts near the volcano apex exhibit slopes of 30–60 mV dec^−1^ consistent with a pre-equilibrium step before the RDS; IrO_2_ and RuO_2_ in acid typically show 40–60 mV dec^−1^; many oxide catalysts on the left branch of the OER volcano exhibit slopes of 90–120 mV dec^−1^ diagnostic of the O* → HOO* step as RDS [15,23]. The OER Tafel slope is further complicated by potential-dependent changes in the surface oxidation state of the metal center during the measurement window, which can produce apparent slopes that carry no single mechanistic assignment.

### 4.2. Potentiodynamic Extraction and Systematic Underestimation of b

**Figure 2:**
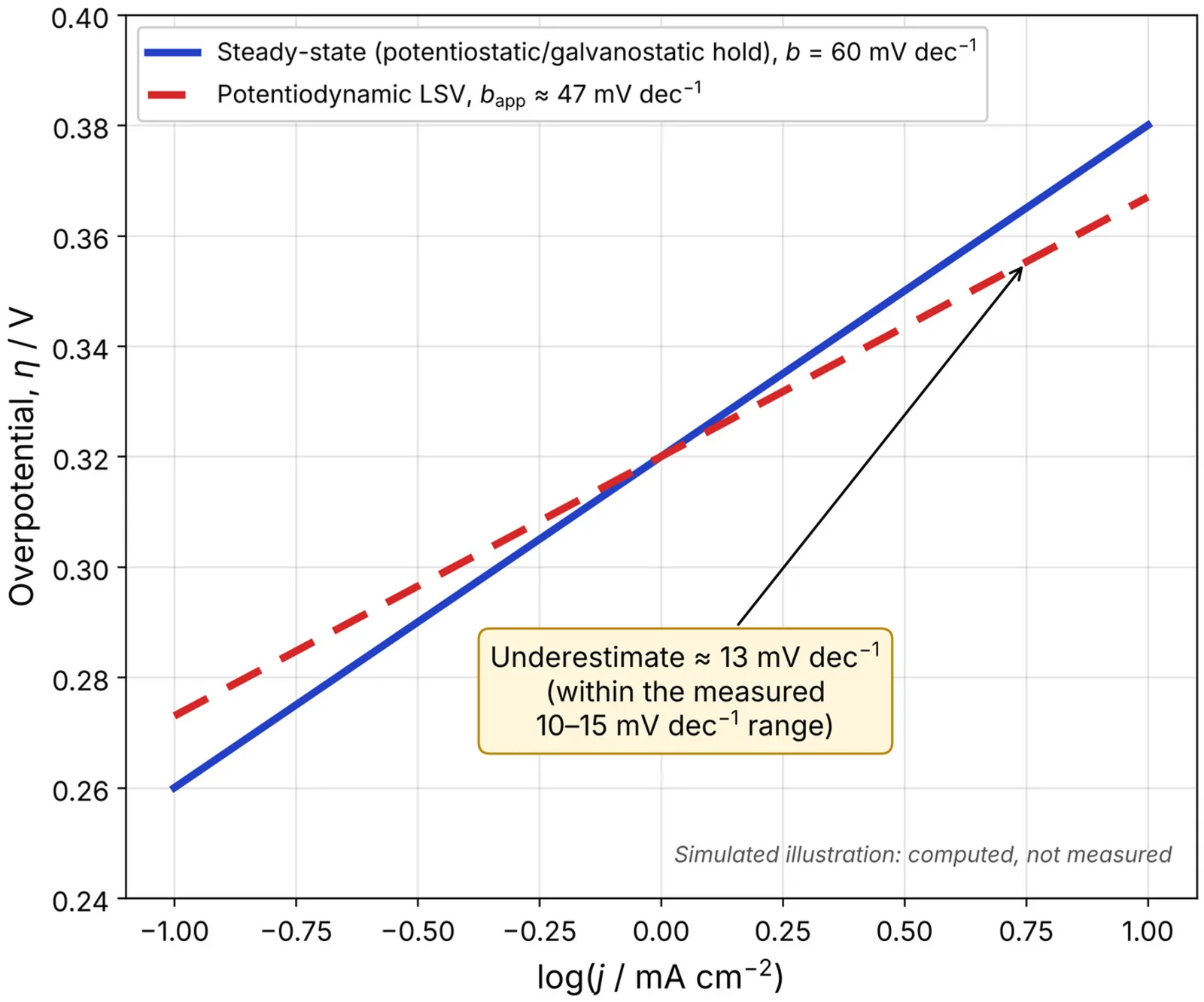
Simulated Tafel plots illustrating the underestimation of Tafel slope by potentiodynamic extraction relative to steady-state measurement for an OER catalyst. Both lines are computed from Equation (2) with illustrative parameters and are not measured data: the steady-state line uses *b* = 60 mV dec^−1^ and the potentiodynamic line *b*_app_ = 47 mV dec^−1^. The 13 mV dec^−1^ gap lies within the 10 to 15 mV dec^−1^ range measured on an activated cobalt foil in 1 M KOH [24]; under high mass transport, the gap approaches zero [25].

The most consequential error in Tafel slope reporting, though its severity depends heavily on measurement conditions, is the extraction from linear-sweep voltammograms (LSVs) acquired potentiodynamically rather than from true steady-state measurements. In a potentiodynamic LSV, the total measured current density at any potential comprises a Faradaic component *j*_F_ and a non-Faradaic capacitive charging component *j*_C_:

*j*_total_ = *j*_F_ + *j*_C_ = *j*_F_ + C_dl_ν (3)

where C_dl_ is the double-layer capacitance, and ν is the scan rate (V s^−1^). At the low current densities characteristic of the Tafel region (typically 0.1–5 mA cm^−2^ for laboratory-scale catalysts), *j*_C_ can represent a substantial fraction of *j*_total_ even at scan rates as low as 1–5 mV s^−1^. A charging current that is constant across the sweep would, if anything, raise the apparent slope at low current density. The lower potentiodynamic slopes reported experimentally are more consistent with Faradaic currents that have not relaxed to steady state, including surface redox charging that grows with potential. Voiry et al. recommend that Tafel slopes be extracted only from steady-state polarization data acquired by galvanostatic or potentiostatic step methods with sufficient equilibration time at each point [15]. Anantharaj and Noda show that, of the sweep rates they compared, only 0.1 mV s^−1^ approached the steady-state slope (Figure 2) [17]. The error increases monotonically with scan rate: even the 10 mV s^−1^ scan rate specified in the McCrory protocol is not steady-state for catalysts with C_dl_ > 0.5 mF cm^−2^, which includes most high-surface-area non-precious metal catalysts [14,15]. Independent primary data confirm the same direction and rough magnitude of this error. For an activated cobalt foil OER catalyst in 1 M KOH, steady-state chronoamperometric sampling gave a Tafel slope of 60 mV dec^−1^ at low overpotential and 120 mV dec^−1^ at high overpotential, with an exchange current density of 5.9 × 10^−5^ mA cm^−2^; potentiodynamic LSV at standard scan rates (0.001–0.010 V s^−1^) returned slopes of only 45–50 mV dec^−1^, 10–15 mV dec^−1^ low, and even the slowest scan rate tested (0.0001 V s^−1^) still gave an exchange current density an order of magnitude below the steady-state value [24]. On the primary data available, the underestimation is therefore about 10 to 15 mV dec^−1^ under typical screening conditions and close to zero under well-controlled mass transport (below). The discrepancy is not universal, however, and the conditions under which it appears matter for how the artefact should be reported. Under high-mass-transport conditions (4000 rpm rotating disk, low catalyst loading, high hydroxide concentration), potentiodynamic, chronoamperometric, and impedance-derived Tafel slopes for OER on NiFeOOH converged to within 1.3 mV dec^−1^ of each other (31.1, 30.4, and 31.7 mV dec^−1^, respectively) [25]. The same study found no kinetically meaningful horizontal Tafel region for alkaline HER or CO_2_ reduction under commonly used conditions, regardless of the technique applied [25]. Read together, these results indicate that the potentiodynamic-extraction artifact is most severe for high-capacitance, self-oxidizing catalysts measured at high loading and low mass transport, conditions typical of unoptimized laboratory screening, and can be substantially suppressed by loading and mass-transport control that is itself rarely disclosed.

### 4.3. Minimum Reporting Requirements for Tafel Slope

The minimum protocol for reliable Tafel slope reporting consists of four requirements. First, the current acquisition must be steady-state, with a stability criterion of less than 1% variation over the final 30 s of a 120-s potentiostatic hold at each potential step [15]. If potentiodynamic collection is unavoidable, the lowest practical scan rate should be used, since in Anantharaj and Noda’s comparison only 0.1 mV s^−1^ approached the steady-state slope [17], and non-Faradaic background current must be subtracted by measuring *j*_C_ in the non-Faradaic potential window and removing it from the total current at each potential [15]. Second, the overpotential range over which the linear Tafel fit is performed should span at least 150 mV [15], though this criterion is difficult to satisfy for OER catalysts whose Tafel region curves at low overpotentials due to coverage effects and at high overpotentials due to mass transport limitations; the precise range used must be reported. Third, the exchange current density *j*_0_ should be extracted by linear extrapolation of the Tafel fit to η = 0 and explicitly reported alongside *b*, since *j*_0_ provides the intrinsic activity benchmark required for volcano plot placement. For OER catalysts with Tafel slopes exceeding 60 mV dec^−1^, the extrapolation distance from the measurement window to η =  0 introduces uncertainty of 50–150 mV in *j*_0_, which should be acknowledged [15,22]. Fourth, the iR-corrected potential must be used throughout; the value of *R*_u_ and the compensation method applied must be disclosed [26,27].

## 5. Electrochemically Active Surface Area: Double-Layer Capacitance Methods and Normalization Artefacts

### 5.1. ECSA Determination by Double-Layer Capacitance

**Figure 3:**
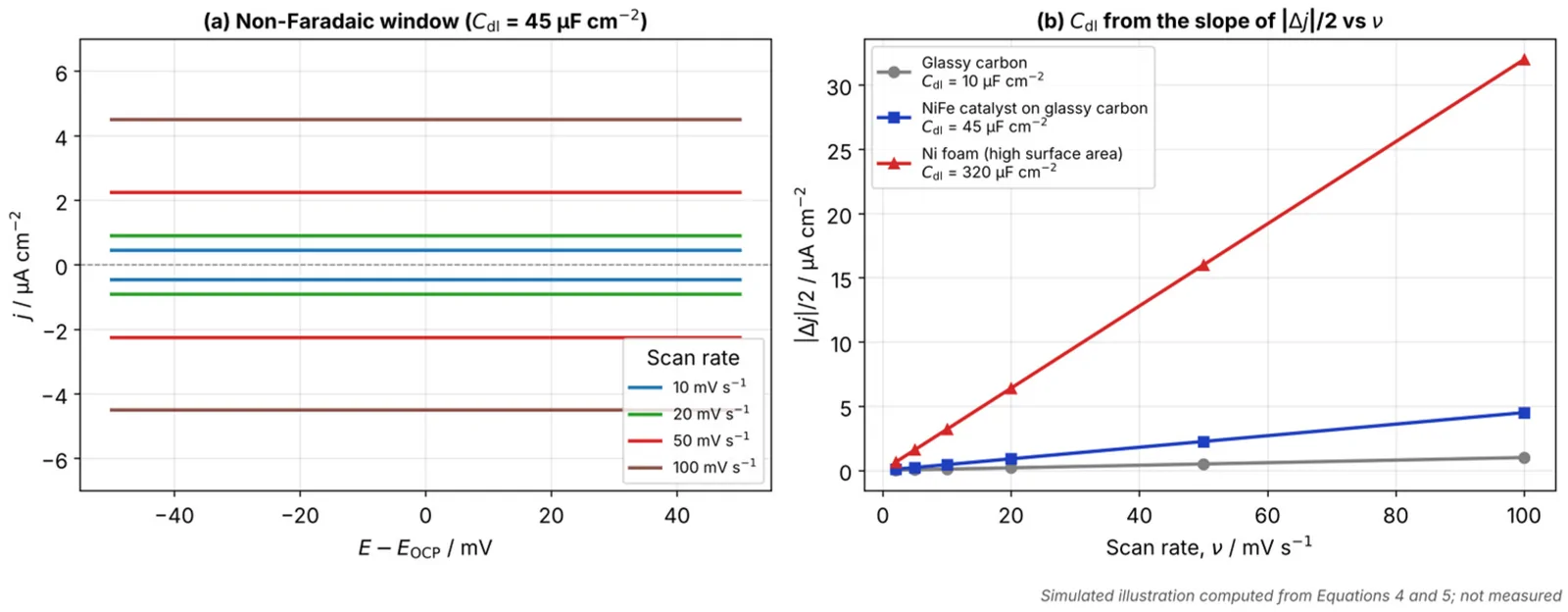
Simulated illustration of ECSA determination by the double-layer capacitance method, generated from Equation (4) with assumed C_dl_ values chosen to represent typical orders of magnitude; not measured data. (**a**) Non-Faradaic cyclic voltammograms with rectangular profiles at scan rates of 10–100 mV s^−1^ in the non-Faradaic potential window. (**b**) |Δ*j*|/2 versus scan rate for three electrode types: bare glassy carbon (C_dl_ ≈ 10 μF cm^−2^), NiFe/glassy carbon catalyst (C_dl_ ≈ 45 μF cm^−2^), and Ni foam (C_dl_ ≈ 320 μF cm^−2^). The slope of each regression gives C_dl_; ECSA = C_dl_/C_s_ where C_s_ = 40 μF cm^−2^ (smooth oxide reference) [14].

The ECSA is the quantity required to convert geometric current density to intrinsic specific activity, and its determination by the double-layer capacitance method is the most widely applied approach in the benchmarking literature (Figure 3). The method is based on the relationship:

*j*_C_ = C_dl_ν (4)

A series of cyclic voltammograms at scan rates of 5–100 mV s^−1^ in a potential window free of redox transitions yields a linear plot of *j*_C_ versus ν, the slope of which gives C_dl_ (mF cm^−2^). The ECSA is then:

ECSA = C_dl_/C_s_ (5)

where C_s_ is the specific capacitance per unit real surface area. The standard value used in the benchmarking community is C_s_ = 40 μF cm^−2^ for smooth oxide surfaces in aqueous electrolyte, a value derived from measurements on flat noble metal electrodes [14]. and attributed to the foundational analysis by Trasatti and Petrii [28].

### 5.2. Systematic Sources of Error and the ±1 Order of Magnitude Uncertainty

The C_dl_/ECSA method carries at least four structural limitations that render its output an order-of-magnitude estimate at best. **First**, the assumed C_s_ = 40 μF cm^−2^ is appropriate for smooth oxide surfaces but is not a constant; it varies with electrolyte, concentration, and oxide surface composition [14]: the double-layer structure at a NiOOH surface in 30 wt% KOH is fundamentally different from that at an IrO_2_ surface in 0.1 M H_2_SO_4_, yet the same Cs is routinely applied to both. Second, for porous and nanostructured electrodes (the majority of high-performance catalysts in the recent literature), the assumption that all electrode surface contributes equally to the measured C_dl_ is violated because interior pore surface is accessed by ionic diffusion with a frequency-dependent response: at sweep rates of 5–100 mV s^−1^, only the outer surface and shallow pore interior are fully accessed, causing Cdl to underestimate the true accessible surface, as the NiFe LDH evidence below shows. Third, the non-Faradaic potential window for OER catalysts must avoid the M^2+^/M^3+^ redox transition, but for many Ni- and Co-based catalysts this transition is broad, so the measured C_dl_ carries a Faradaic contribution that inflates the apparent ECSA, as the iridium oxide evidence below shows. Fourth, and specific to the alkaline NiFe-OOH system central to this manuscript, the C_dl_ measured before electrochemical conditioning is not the same surface as after Fe incorporation and activation: the reconstructed NiFeOOH phase has different surface chemistry, charge, and capacitance from the precatalyst NiOOH. ECSA values calculated from pre-conditioning Cdl measurements do not reflect the surface present during steady-state OER catalysis [29], which is why Anantharaj and Noda recommend repeating these measurements after activation [17].

McCrory et al. acknowledged these limitations and assigned at least ±1 order of magnitude uncertainty to all ECSA values in their original benchmarking dataset, a caution they retained when extending the same protocol to a substantially larger set of 44 HER and OER catalysts two years later [14,30]. The direct consequence is that ECSA-normalized specific activities that differ by less than one order of magnitude between two catalysts cannot be used as the sole basis for claiming intrinsic superiority: the measurement uncertainty alone spans the claimed difference. Anantharaj and Noda caution that double-layer capacitance is an indirect measure of surface area that cannot alone justify an activity trend, and recommend reading it alongside charge-transfer resistance [17]. Independent studies confirm each of these failure modes with quantitative data on different catalyst systems. For iridium oxide, aqueous-electrolyte capacitance showed no linear correlation whatsoever with independently measured Brunauer–Emmett–Teller (BET) surface area across ten samples of varying crystallinity; pseudocapacitance accounted for more than 90% of the total measured capacitance in the least crystalline, pristine sample, and normalizing the same activity data by total capacitance versus by the pseudocapacitance-isolated electrical double-layer (EDL) capacitance produced opposite trends with respect to crystallinity [31]. For NiFe layered double hydroxide, the catalyst system central to this manuscript, non-Faradaic cyclic voltammetry-derived double-layer capacitance was confirmed to badly underestimate the electrochemically accessible area because the material is non-conductive in the non-Faradaic window; switching to electrochemical impedance spectroscopy (EIS)-derived Faradaic capacitance gave a value roughly eight times larger (15.5 vs. 2.0 F s1−α) on the same electrode [32]. At the level of the underlying cyclic voltammetry measurement itself, neglecting proper selection of the measurement window alone was shown to cause a 36% underestimation of double-layer capacitance, and fitting real, non-ideal electrode data to the standard linear ideal-capacitor model rather than an allometric model caused a further 32.6% deviation from the independently EIS-verified value [33]. None of these errors are specific to one material system; together they suggest that the ±1 order of magnitude uncertainty McCrory assigned to ECSA may represent a floor rather than a ceiling for non-precious, non-conductive, or amorphous catalyst layers; none of the three studies cited here independently re-derives McCrory’s bound, so this remains an inference from convergent failure modes rather than a directly measured wider bound.

## 6. Turnover Frequency: Site Density Determination and the Operando Restructuring Problem

### 6.1. Definition and Calculation

The TOF is the number of catalytic events per active site per unit time at a defined overpotential:

TOF = *j*/(*n*_e_ × *F* × Γ_sites_) (6)

where *j* is the current density (mA cm^−2^), *n*_e_ is the number of electrons transferred per catalytic event (2 e^−^ per H_2_ molecule for HER or 4 e^−^ per O_2_ molecule for OER), and Γ_sites_ is the surface density of active sites (mol cm^−2^). A critical convention that must be disclosed is whether TOF is reported per molecule (O_2_ or H_2_) or per electron transferred: the two definitions differ by a factor of 4 for OER and a factor of 2 for HER, so the convention must be stated with every reported value. When reported per geometric area with *j*_geo_, TOF conflates intrinsic activity with the loading-dependent roughness factor and is not a material property. When reported per total metal atom, TOF is a lower bound on the true per-site rate because it assumes all metal atoms are active, which is never true for bulk catalysts.

### 6.2. Site Density Determination Methods

Three methods are commonly used to determine Γ-sites. Electrochemical integration of the surface redox transition peak (for example, the Ni^2+^ → Ni^3+^ anodic wave for NiOOH-based catalysts) provides an estimate of surface-accessible redox-active metal centers, but is subject to incomplete transition at fast sweep rates and overlap with the OER onset at high loadings [14,17]. Inductively coupled plasma optical emission spectroscopy (ICP-OES) analysis of the dissolved catalyst provides the total metal atom count without the accessibility constraint but gives a lower bound on TOF by over-counting inactive bulk atoms. BET surface area measurement and geometric site density estimation from crystallographic data provide a third approach, applicable when the active site is well-defined and crystallographically characterized, but both methods fail when the active site is an amorphous surface species, as is universally the case for reconstructed (oxy)hydroxide OER catalysts. Anantharaj and Noda warn against assuming that all loaded atoms are active and against geometric or mass-normalized TOF [17]; the two- to three-order-of-magnitude shift between site-counting bases is documented in [8]. Table 3 shows what this looks like on one material rather than as a general claim. A kinetic probe study of iron single-atom Fe-N-C oxygen reduction catalysts benchmarked a hydroquinone oxidation method against three independently established site-counting techniques on the same set of catalysts, introduced in Section 1. No two methods agree, and the disagreement is not small: even the two most concordant pair, hydroquinone oxidation and CO chemisorption, differ by a mean percentage error of 26%.

Table 3 comes from Fe-N-C oxygen reduction catalysts, not HER or OER materials, and its methods do not all probe the same population of sites: CO chemisorption and NO stripping count accessible Fe sites, while Mössbauer spectroscopy reports bulk Fe speciation. These probes are specific to atomically dispersed Fe–N_x_ sites, and none of them can be applied to reconstructed NiFe or Co oxyhydroxide OER catalysts, for which the redox-peak, ICP-OES, and BET approaches above are used instead. We use Table 3 as a best case. Single-atom Fe-N-C sites are among the most structurally defined active sites in electrocatalysis, yet the methods still disagree by 26 to 53%. Reconstructed Ni and Co oxyhydroxides, with amorphous sites that change under bias (Section 6.3), give little reason to expect closer agreement. We are not aware of an equivalent multi-method comparison for OER catalysts, however, and treat the transfer as an inference. The OER-specific evidence is the two- to three-order-of-magnitude shift between redox-peak and total-metal TOF bases [8] and the film-thickness dependence of TOF in NiFe oxyhydroxides [12].

### 6.3. The Operando Restructuring Problem

The most fundamental limitation of TOF as a benchmarking metric for OER catalysts is the ubiquitous and often complete transformation of the catalyst precursor during electrochemical conditioning. It is well established that Ni- and Co-based oxides, phosphides, sulfides, and metal–organic frameworks spontaneously reconstruct to form NiOOH or CoOOH surface phases under anodic OER conditions [29,34]. Operando evidence corroborates this across precursor classes. For atomically dispersed Ir stabilized on a nanoporous metal phosphide, operando X-ray absorption spectroscopy shows the metal–P coordination shell collapsing as a metal–O shell forms under anodic potential, evidencing conversion of the support to an oxyhydroxide surface [35]. For a NiCo bimetallic sulfide, in situ electron microscopy resolves lattice sulfur being displaced by oxygen from the electrolyte before the particle surface reconstructs into an amorphous oxyhydroxide shell up to 127 nm thick [36]. For a cobalt-iron metal–organic framework, X-ray absorption spectroscopy tracks a two-step transformation, first to a Co(II)/Fe(III) hydroxide and then, under anodic potential, to a Co(Fe)OOH phase confirmed as the catalytically active species [37]. The electrochemically active species at the end of a stability test is therefore not the material characterized by XRD, transmission electron microscopy (TEM), and ICP before the experiment, and the site density calculated from precatalyst characterization is not the site density of the species responsible for the observed OER activity. Reporting TOF based on pre-reaction site characterization for a catalyst known to undergo operando reconstruction is physically inconsistent: it describes the activity per site of a material that is not present during catalysis. The rigorous resolution requires operando characterization (in situ XAS, operando Raman spectroscopy, or 18O isotope labeling with differential electrochemical mass spectrometry (DEMS)) to determine the true active species and its surface density under catalytic conditions. [34] Where such techniques are not available, the minimum acceptable practice is post-reaction characterization (XRD, X-ray photoelectron spectroscopy (XPS), TEM, ICP) to document the final state of the electrode, accompanied by a clear statement that the reported TOF describes the activity per site of the reconstructed species rather than the nominal precatalyst. TOF values attributed to a precatalyst that is not present during catalysis should not be used as evidence of intrinsic activity [34].

## 7. Faradaic Efficiency: Measurement Protocols and Systematic Errors

### 7.1. Definition and Significance

Faradaic efficiency (FE) is defined as the fraction of charge passed that is converted to the target product (H_2_ at the cathode or O_2_ at the anode) relative to the theoretical yield calculated from Faraday’s law:

FE = (*n*_meas_ × *n*_e_ × *F*)/*Q*_total_ (7)

where *n*_meas_ is the measured moles of H_2_ or O_2_, *n*_e_ = 2 (HER) or 4 (OER), and *Q*_total_ is the total charge passed. A Faradaic efficiency below 100% for HER indicates diversion of current to competing cathode reactions or unrecovered dissolved H_2_. For OER, FE < 100% carries a mechanistically specific implication: it indicates that the anodic current is partially diverted to peroxide (H_2_O_2_) formation or to catalyst oxidation reactions that do not yield O_2_. Peroxide formation is specifically a signal of AEM mechanism failure: it arises when HOO* desorbs from the surface as H_2_O_2_ rather than undergoing the fourth proton–electron transfer step of the associative OER mechanism to yield O_2_. FE is therefore simultaneously a product-selectivity metric and a mechanistic indicator, and a decline in FE over time signals the emergence of competing reactions associated with catalyst degradation [14,15].

### 7.2. Measurement Methods and Their Systematic Errors

Gas chromatography (GC) with a calibrated thermal conductivity detector is the most accurate method for FE determination, providing absolute quantification with uncertainties below 3% when properly calibrated. [15] The principal systematic errors in GC-based FE measurement are: gas dissolution in the electrolyte before collection, particularly significant for H_2_ in alkaline electrolytes at low current densities, where the dissolved fraction can represent 20–50% of total production; [15] gas crossover through the separator between anodic and cathodic compartments; and incomplete collection of gas from the headspace at low current densities where gas evolution is slow relative to headspace diffusion. The water-displacement method introduces greater uncertainty because the volume of gas displaced depends on atmospheric pressure, electrolyte temperature, and gas solubility, all of which must be measured and corrected independently. For H_2_, whose solubility in water is approximately 1.6 × 10^−3^ mol L^−1^ atm^−1^ at 25 °C, the dissolved fraction is non-negligible at current densities below 20 mA cm^−2^ and causes systematic underestimation of FE. The rotating ring-disk electrode (RRDE) method provides real-time, in situ FE measurement by collecting O_2_ generated at the disk and detected by a concentric Pt ring [14,30]. The collection efficiency ***N*** of the RRDE geometry must be determined experimentally using a stable outer-sphere redox couple before OER experiments; use of the theoretical *N* without experimental calibration introduces systematic errors of up to 15% in the reported FE [14,30]. McCrory et al. employed RRDE-based FE measurement as a required component of their benchmarking protocol for OER catalysts, providing a direct cross-check that anodic current arises entirely from O_2_ evolution [14]. Voiry et al. recommend FE measurements at multiple current densities and emphasize that disclosure of the measurement method and all correction factors applied is a minimum reporting requirement [15]. In the context of industrial-scale evaluation, sustained current densities and long operating durations make single-timepoint FE values insufficient on their own. Kempler and Nielander set out thermodynamic criteria for when an FE measurement should be considered mandatory rather than optional: whenever more than one product is thermodynamically accessible at the applied potential, whenever contaminant or crossover species could compete for current, or whenever any electrode or support component is not stable under the applied potential and electrolyte [18]. They also draw a distinction the OER benchmarking literature rarely makes explicit: Faradaic efficiency is an integrated measure over a sampling period, whereas current efficiency describes instantaneous selectivity at a single moment, and the two terms are routinely conflated in practice. Reported FE values should carry error bars derived from at least three independent measurements rather than being presented as single figures [18].

## 8. Applying the Framework: A Worked Case Study

The preceding sections diagnose non-comparability in the abstract. This section tests the diagnosis against real numbers. Two literature arms were compiled: one for OER, one for HER. Every value used came from each primary source’s experimental and results sections. The OER arm covers plain, minimally-modified NiFe (oxy)hydroxide or LDH catalysts in alkaline media; the HER arm covers commercial Pt/C under the same conditions. Both arms report η_10_ where available, in 1 M KOH, at room temperature unless otherwise noted. Full source-level details for both arms are provided in the Supporting Information (Tables S1 and S2). **Search and selection process.** Candidates for both arms were identified primarily by tracing citation chains through the mechanistic and methodological literature already surveyed in Section 1, Section 2, Section 3, Section 4, Section 5, Section 6 and Section 7, rather than by a single database query; for the OER arm, four additional candidates were located through one review article [38], of which one (Louie and Bell [39]) contributed a comparable η_10_ data point after direct verification against the primary source. Three named candidates were located but could not be confirmed against a primary source at the time of writing (two OER: a report in RSC Advances and an unconfirmed report in the Journal of Fuel Chemistry and Technology; one HER: a patent reporting 59 mV for Pt/C in alkaline electrolyte) and are excluded from both arms’ totals and from Tables S1 and S2, a status kept visible rather than silently dropped. Inclusion required that the reported value trace to the source paper’s own experimental or results section; a value taken from an abstract, a review’s summary table, or a citation chain without independent verification was excluded regardless of how often it recurs elsewhere in the literature. Values reported on a different basis, η_50_/η_100_ rather than η_10_ [40,41,42], a loading series rather than a single value [43], or a non-standard electrolyte [39,44,45,46], were retained in the qualitative discussion (Section 8.1, Section 8.2 and Section 8.3) but excluded from the matched-basis summary statistics in Table 4. Applying these criteria yielded 11 checked OER sources [39,40,41,42,44,45,47,48,49,50,51] and 7 checked HER sources [43,46,52,53,54,55,56], of which 5 OER and 5 HER sources met the further, stricter matched-basis criterion (η_10_, 1 M KOH, room temperature) underlying Table 4. This is a targeted, hand-verified compilation rather than a systematic database search run against a fixed keyword string; that trade-off is revisited in Section 8.5.

### 8.1. The OER Arm: An Already-Unstable Material Gets an Unstable Number

Restricted to standard conditions, 1 M KOH, room temperature, reported η_10_ for nominally the same class of catalyst spans 134 mV to 372 mV, a near-threefold range [47,48], with an undoped NiFe-LDH source reporting 175 mV under the same conditions [49]. Three entries sit further out once non-standard conditions are counted rather than excluded: Dionigi et al. report 348 mV in 0.1 M KOH [44], Wang et al. report 514 mV for NiFe electrodeposited on stainless steel, also in 0.1 M KOH [45], and Louie and Bell report 280 mV at 40% Fe content, also in 0.1 M KOH [39]. A useful internal control comes from Dai et al., who report four η_100_ values for the same catalyst (NiFe LDH/NF) under four electrolyte conditions: 233 mV in 1 M KOH, 180 mV in 6 M KOH at 60 °C, 243 mV in alkaline artificial seawater, and 280 mV in alkaline natural seawater [40], a 100 mV swing from electrolyte alone with no change to the catalyst itself. A second internal control, at η_10_ rather than η_100_, comes from Tang et al.: NiFe_0.5_(OH)_x_ at 265 mV, measured in the same paper alongside Ni(OH)_x_ (389 mV), Fe(OH)_x_ (430 mV), and IrO [4] (272 mV), all in 1 M KOH on the same setup [50]. A further source of non-comparability is independent of substrate, electrolyte, or reconstruction state: some source papers avoid η_10_ entirely. Two reports on NiFe hydroxide films state that a Ni^2+^/Ni^3+^ oxidation peak near 1.35 to 1.40 V distorts the linear sweep voltammetry curve at low current density, and standardize on η_50_/η_100_ instead [41,42]. Any table that forces every source into an η_10_ column either drops legitimate sources or silently misreads a distorted point on their curves. A separate trap appears within a single paper rather than across papers. Jeon et al. report two candidate η_10_ values for what is effectively the same optimization campaign: the abstract’s headline number (NiFe/NiO-130, η_10_ = 245 mV, from the concentration-optimization stage) is not the paper’s best result. After a second-stage Ni:Fe ratio optimization at fixed concentration, NiFe/NiO-20(130) achieves η_10_ = 237 mV [51]. Because our inclusion rule takes values from the results section, Table 4 uses 237 mV. Neither value is flagged in the abstract as superseding the other, so which one gets extracted depends on how far into the paper the extractor reads.

### 8.2. The HER Arm: The Reference Material Is Not a Reference Value

Pt/C functions as the field’s de facto reference point, but the values reported for it show the same non-comparability documented above for novel catalysts. Across independently verified sources, all reporting commercial Pt/C in 1 M KOH at approximately 10 mA cm^−2^, η_10_ ranges from 33 mV (Kweon et al. [52]) to 93.4 mV (Samanta et al. [53]), a 2.8-fold spread, and Tafel slope ranges from 30 mV dec^−1^ (Bekmyrza et al. [54]) to 105 mV dec^−1^ (Sadeghi et al. [55]), a 3.5-fold spread. This is the same benchmark material, reported by independent groups, none of whom set out to characterize Pt/C; in every case it appears as the comparator against which their own novel catalyst is judged. The disclosure pattern is the more damning finding. Wan et al. disclose Pt mass loading directly across a full loading series, 5 to 200 μg cm^−2^, and show Tafel slope for the same commercial Pt/C material running from 33 to 66 mV dec^−1^ across that range, and from 63 to 211 mV dec^−1^ at fixed loading depending solely on the overpotential window used for fitting [43]. Wan et al. trace this to two competing effects: locally trapped H_2_ in the catalyst layer suppresses current at higher overpotential, inflating the apparent slope, while a non-negligible hydrogen oxidation current partially cancels HER current at lower overpotential, deflating it. They propose Butler–Volmer fitting as a partial fix, though it holds less well for transition-metal-decorated Pt surfaces [43]. Zhang et al. also disclose loading directly, but it is not held fixed across their two reported conditions: 0.050 mg cm^−2^ Pt on a glassy carbon disk in 1 M KOH at room temperature (η_10_ = 39 mV), versus 0.10 mg cm^−2^ Pt on carbon cloth in 5 M KOH at 60 °C (η_10_ = 32.1 mV); loading, electrolyte concentration, temperature, and substrate all change together [56]. Sadeghi et al. allow indirect reconstruction: 1 mg of 20 wt% commercial Pt/C dispersed to a total ink loading of approximately 1 mg cm^−2^ on nickel foam implies an actual Pt mass loading of 0.20 mg cm^−2^; their Pt/C comparator reaches η_10_ = 83 mV, Tafel slope 105 mV dec^−1^, in 1 M KOH, with no iR correction stated as negligible at 11.7 Ω [55]. Bekmyrza et al. also require reconstruction rather than stating Pt mass outright: their Pt/C comparator was prepared “following the same procedure” as their own catalyst, a 1.0 mg cm^−2^ total ink loading on nickel foam at 40 wt% Pt, implying 0.40 mg cm^−2^ actual Pt, double Sadeghi et al.’s reconstructed value; it reaches η_10_ = 48 mV, Tafel slope 30 mV dec^−1^, in 1 M KOH [54]. The remaining entries in this arm give no loading information at all, including Kweon et al., whose ruthenium on multi-walled carbon nanotube (Ru@MWCNT) paper’s Pt/C comparator (33 mV, 43 mV dec^−1^, 1 M KOH) is the low end of the arm’s spread [52]. A catalyst whose activity is known to scale with mass loading is, in most cases, being used as a benchmark with an undisclosed, only indirectly reconstructable, or inconsistently reported mass. Zhang et al.’s second condition and one patent are kept outside the core table for non-standard electrolytes. Cui and Chen’s metal-hydrogen battery patent reports 71 mV for Pt/C in approximately 30 wt% KOH, roughly 5.3 M, again concentrated electrolyte, with no equivalent 1 M KOH figure given [46].

### 8.3. Tafel Slope Is Not a Fixed Property Either

The methodology study introduced above is the anchor for this point as well: the same Pt/C material, in 1 M KOH, gives a Tafel slope running from 33 to 66 mV dec^−1^ across loadings from 5 to 200 μg cm^−2^, and from 63 to 211 mV dec^−1^ at fixed loading depending solely on the overpotential window used for fitting [43]. Neither loading nor fitting window is a hidden variable in the electrochemical sense: both are chosen by the experimenter, and both are reported here well enough for a reader to reproduce, which is precisely what makes the paper useful as a cautionary anchor rather than an outlier to be excluded.

### 8.4. Reading the Two Arms Together

The OER arm and the HER arm fail for overlapping yet distinct reasons. The OER arm’s headline spread likely reflects a combination of genuine catalyst variation and substrate and electrolyte choices layered on top of it, compounded by a subset of papers that avoid η_10_ altogether and by multi-stage optimization papers that leave more than one candidate headline number in the same abstract. The HER arm’s spread is driven by the same measurement platform and data-processing choices this Perspective catalogs in Section 3, Section 4, Section 5, Section 6 and Section 7, applied to a catalyst that is not even the subject of the study reporting it. That a reference material accumulates the same non-comparability as the novel catalysts it is meant to benchmark is, on its own, sufficient evidence that the field’s central benchmarking convention needs the disclosure standard this Perspective proposes. That combined pattern is worth putting a number to, not just a range. Restricting each arm to sources reporting η_10_ in 1 M KOH at room temperature on the same basis five OER sources (Chen, Geerts, Tang, Jeon, Li et al. [47,48,49,50,51]) and five HER sources (Zhang, Sadeghi, Bekmyrza, Kweon, Samanta et al. [52,53,54,55,56]) the OER arm has a mean of 237 mV, a median of 237 mV, a standard deviation of 92 mV, and a coefficient of variation of 39% (Table 4). The HER arm, all reporting the same commercial catalyst class, has a mean of 59 mV, a median of 48 mV, a standard deviation of 27 mV, and a coefficient of variation of 46%. With five sources per arm, neither coefficient of variation is precise: bootstrap 95% intervals are 14 to 52% for the OER arm and 11 to 55% for the HER arm, so the two cannot be distinguished. CV also scales inversely with the mean, and a fixed methodological offset of 20 to 30 mV weighs far more on a 59 mV Pt/C mean than on a 237 mV NiFe mean. Pt/C is, moreover, an HER reference and not a calibrant for OER catalysts, so the two arms are best read separately. The informative quantity is therefore the absolute spread of the reference material itself. Commercial Pt/C, where catalyst-to-catalyst variation should be small, spreads by 60 mV across five matched-basis reports (SD 27 mV). Part of that spread may reflect differences between commercial products (20 and 40 wt% Pt appear in this set), which is itself a disclosure gap; the remainder is methodological. Sources reporting a different basis η_50_/η_100_ rather than η_10_ [40,41,42], a loading series rather than a single value [43], or a non-standard electrolyte [39,44,45,46] are excluded from this calculation rather than folded in on an assumed equivalence, for the same reason argued against elsewhere in this Perspective.

The source papers themselves are not making the comparability failure. Where a paper offers a causal story for its catalyst’s performance, that story is invariably about the catalyst: Louie and Bell tie OER activity to Fe-induced formation of Ni^4+^ centers [39]; Dionigi et al. trace it to an anodic phase transition that forms O-bridged Fe–M reaction centers [44]; Dai et al. attribute it to nanosheet morphology and Ni–Fe electronic synergy, backed by operando Raman [40]. These are substantive, evidenced claims, and nothing here disputes them. But none of these claims concerns loading, electrolyte concentration, or substrate. A paper explaining why a catalyst works has no reason to flag that its headline number moves by 100 mV in a different electrolyte. The two conversations, mechanism and measurement, run in parallel without ever intersecting, and the second is where this Perspective’s six-stage pipeline operates.

### 8.5. Limitations

Both arms are bounded, not exhaustive. Three further candidates, two OER reports and one HER patent reporting 59 mV for Pt/C in alkaline electrolyte, could not be verified against a primary source and were excluded. Four primary sources were identified via a review article [38] but, following the same rule used throughout this arm, values from a review’s summary table were not used until checked against the original. All four have since been checked directly. Trotochaud et al. and McCrory et al. are mechanistic and methodological references, respectively, rather than sources of a comparable η_10_ data point: Trotochaud et al. report turnover frequency as a function of Fe content and film thickness rather than overpotential at fixed current density [12], and McCrory et al. establish the benchmarking protocol this Perspective’s Section 3 discussion draws on [14]. Smith et al. report onset potential and Tafel slope for amorphous mixed-metal oxide films rather than η_10_, and so, like the two J. Alloys Compd. papers discussed above, do not contribute a comparable data point to this arm [57]. Louie and Bell do report η_10_ and have been incorporated into the flagged, non-standard-electrolyte group in Section 8.1 [39].

### 8.6. When Existing Metrics Remain Useful

Everything above is a claim about non-comparability across sources, not a claim that these five metrics are unfit for use within a single, controlled comparison. Three settings, already implicit in the framework above, are where the standard metrics continue to do real work. **Same-lab ranking.** A same-lab ranking claim is exposed only to stages 3 and 4 (Table 1): synthesis, testing platform, iR compensation practice, and normalization basis are held fixed across the catalysts being compared, so an uncorrected or non-ECSA-normalized η_10_ can still validly rank catalysts against each other, provided the same choices are applied uniformly across the set. What this Perspective disputes is carrying that number across a lab boundary, not using it within one. **Standardized benchmarking protocols.** When the full McCrory protocol, glassy carbon substrate, specified electrolyte, RDE voltammetry at fixed rotation and scan rate, is followed as originally specified, ten independently tested earth-abundant OER catalysts converged to an 80 mV overpotential window (Section 3.2), consistent with the interpretation that much of the spread visible in the wider literature is a protocol artifact rather than a real difference in intrinsic activity [14]. A shared protocol, applied and reported in full, substantially recovers comparability. The same logic extends to industrial and standards-body testing: when one facility or protocol fixes current density, electrolyte, and cell design across vendors, a cross-vendor comparison inherits the same protection that homogeneous testing gives lab-scale rankings, and the burden reduces to stating, once, which choices were held fixed. **η_10_ within its stated conditions.** η_10_ remains informative precisely under the conditions Wei and Xu and Anantharaj and Noda specify: ECSA-normalized rather than geometric current density, loadings matched and disclosed, and electrolyte matched to the application [17,19]. The device-context rationale behind the original 10 mA cm^−2^ choice also still holds for its original purpose: screening-scale comparison at the current densities early-stage catalyst research is actually conducted at, rather than as a proxy for performance at the 200–2000 mA cm^−2^ densities industrial electrolyzers operate at (Section 3.2). None of these three settings is the default condition under which η_10_, Tafel slope, ECSA, TOF, or Faradaic efficiency are currently reported in the literature surveyed in Section 8; that gap, between the conditions under which these metrics are valid and the conditions under which they are actually used, is the problem this Perspective addresses, not a case against the metrics themselves.

### 8.7. Consequences for Mechanistic Interpretation and Catalyst Design

The artifacts catalogued above are not confined to benchmarking tables; the same choices propagate into mechanistic assignment and, from there, into catalyst design. Tafel slope is the primary experimental handle for rate-determining-step assignment (Section 4.1): a slope of 30–60 mV dec^−1^ near the OER volcano apex is read as a pre-equilibrium step ahead of the RDS, 40–60 mV dec^−1^ for IrO_2_/RuO_2_ in acid, and 90–120 mV dec^−1^ on the volcano’s left branch as diagnostic of the O* → HOO* step [15,23]. Potentiodynamic extraction underestimates the steady-state slope by about 10 to 15 mV dec^−1^ under typical screening conditions (Section 4.2). That is enough to move a true 60 mV dec^−1^ slope into the 45 to 50 mV dec^−1^ range, across the boundary between neighboring limiting values [22], so a measurement artefact rather than a change in mechanism can shift an RDS assignment in the published record. It is not, on the primary data available, enough to move a left-branch catalyst at 90 to 120 mV dec^−1^ into the apex range. The same problem reaches the volcano’s activity axis directly. Exchange current density *j*_0_, the standard quantity plotted against a binding-energy descriptor, is extracted by extrapolating the Tafel fit to η = 0; for slopes above 60 mV dec^−1^ this extrapolation alone carries 50–150 mV of uncertainty in *j*_0_ (Section 4.3) [15,22]. Turnover frequency, the metric used interchangeably with *j*_0_ in much of the descriptor literature, carries an independent 2- to 3-order-of-magnitude uncertainty from site-counting convention alone (Section 6), on top of the ±1-order-of-magnitude ECSA uncertainty (Section 5) any per-site normalization inherits. A structure-activity correlation built by comparing TOF or *j*_0_ values drawn from papers that made different, undisclosed choices at any of these stages is fitting a line through points whose vertical scatter includes an unknown, and possibly dominant, contribution from methodology, not exclusively from the descriptor on the horizontal axis. Section 8.2 shows this is not a hypothetical concern: commercial Pt/C, the most common HER volcano anchor point, itself shows a 2.8-fold spread in η_10_ and a 3.5-fold spread in Tafel slope across independently reported values. If the field’s fixed reference point moves by this much across sources, a novel catalyst’s position relative to it is not fixed either. The consequence extends past any single mechanistic claim to how catalysts are selected for further study. Descriptor-based screening, and increasingly data-driven or machine-learning models trained on literature-compiled activity values, treat the reported TOF, *j*_0_, or η_10_ as ground truth for the dependent variable. Where the between-source variance introduced by undisclosed methodology approaches or exceeds the catalyst-to-catalyst variance the model is meant to learn from, exactly the situation Table 4 documents for both case-study arms, a design rule or predicted lead compound built on that dataset is partly fit to reporting practice rather than to intrinsic reactivity. The corrective is the same one this Perspective proposes throughout: the disclosure in Table 5 is what lets a mechanistic or design conclusion be checked against the measurement choices that produced it, rather than taken on faith.

## 9. Toward Next-Generation Electrocatalyst Benchmarking

Table 5 is a starting point, not an endpoint. Closing the gap it documents between the conditions under which η_10_, Tafel slope, ECSA, TOF, and Faradaic efficiency are valid and the conditions under which they are actually reported is a field-level project with at least four concrete directions. Interlaboratory validation, already applied to synthesis reproducibility, is directly extensible to the measurement stage. The 2025 global interlaboratory study discussed in Section 1 used a design-of-experiments approach across ten independent groups to quantify which synthesis parameters actually drove the observed two-order-of-magnitude spread in a NiFe OER catalyst [4]. The same design, applied instead to a single, pre-characterized catalyst measured independently under a shared protocol, would convert the informal uncertainty this Perspective infers from Table 4’s Pt/C statistics, a 27 mV standard deviation on the field’s own reference material, into a directly measured, citable uncertainty budget for each of the five metrics in Table 5. Open, primary-source-verified datasets are the natural complement. Section 8’s case study required tracing each of 18 checked sources back to its own experimental section by hand, and named three further candidates that could not be verified in the time available (Section 8.5). A shared, versioned repository in which each entry carries its primary-source citation alongside the Table 5 disclosure fields would let that verification accumulate across papers and research groups rather than being repeated, unconfirmed, inside each new review’s citation chain. Reporting is the piece journals can act on directly, and fastest. A structured, machine-checkable submission template covering the Table 5 fields, loading and substrate for η_10_, extraction method and fit window for Tafel slope, assumed C_s_ for ECSA, site-counting convention for TOF, measurement method for Faradaic efficiency, could be validated for completeness at submission the same way a data-availability statement already is, without requiring an editor or reviewer to locate each disclosure inside the methods section. Automated extraction tools, including large language models, can plausibly accelerate the verification step this Perspective performed manually in Section 8, but only if the acceleration is applied to checking values against their primary source rather than to propagating a value from a review’s summary table, the exact failure mode Section 8’s search and selection process was built to exclude. Used to flag rather than replace verification, such tools shorten the time between a value being published and being checked, not the standard to which it is checked.

## 10. Conclusions

Across the five metrics examined in this Perspective, the same three magnitudes recur: tens of millivolts, one order of magnitude, and up to three orders of magnitude, and in every case, the source is a specific, identifiable methodological choice, not a flaw in an otherwise canonical technique. Overpotential at 10 mA cm^−2^ inherited a device-context rationale matching neither laboratory screening nor the current densities, two orders of magnitude higher, at which industrial relevance is claimed, and its geometric normalization moves by 40–60 mV with loading alone (Section 3). Tafel slopes extracted potentiodynamically are shallower than their steady-state values by about 10 to 15 mV dec^−1^ under typical screening conditions, enough to blur the boundary between neighboring mechanistic assignments (Section 4). Electrochemically active surface area carries at least a ±1-order-of-magnitude uncertainty under the original benchmarking protocol’s own accounting, before accounting for porous-electrode, reconstruction, and electrolyte-composition effects (Section 5). Turnover frequency inherits whichever of three common site-counting conventions its author chose, shifting the reported value by two to three orders of magnitude, and is further undermined for most Ni- and Co-based OER catalysts by operando reconstruction that leaves the pre-reaction site count irrelevant to the species actually catalyzing the reaction (Section 6). Faradaic efficiency measurement, when reported at all, rarely discloses which of several methods with materially different error profiles was used, or whether the value is a single measurement or an average with reproducibility bounds (Section 7). Section 8 tests this diagnosis against real numbers rather than leaving it at the level of individual artefact reports. Of 11 NiFe oxyhydroxide reports checked against their primary sources, five report η_10_ in 1 M KOH at room temperature, and these span 134 to 372 mV. The other six report in 0.1 M KOH or at 50 or 100 mA cm^−2^, which is itself part of the comparability problem. Commercial Pt/C fares no better: five matched-basis reports span 33 to 93 mV, nearly threefold, with Tafel slopes from 30 to 105 mV dec^−1^ and loading stated directly in only two of seven sources. That the field’s own benchmark material shows the same spread it is meant to rule out is, on its own, evidence that the disclosure standard proposed below addresses a real and current gap, not a hypothetical one. None of these five failures is a reporting error in the colloquial sense: gas chromatography, potentiodynamic Tafel extraction, and geometric normalization are each legitimate techniques routinely chosen by careful researchers. The problem is that the choice among them is rarely disclosed, and Table 1 shows that this choice is not evenly distributed across the kinds of claims a paper makes. A same-lab ranking claim depends most on data-processing and normalization choices (stages 3–4); a cross-literature record claim inherits synthesis reproducibility in full (stage 1) and cannot be rescued by any downstream correction; a mechanistic claim built on turnover frequency inherits whichever site-counting disagreement its author picked (stage 4); an industrial-relevance claim is exposed to test-condition and measurement-platform artefacts (stages 2 and 6) that are negligible at typical screening current densities and large at the densities relevance is actually claimed for. Papers advancing more than one claim type inherit more than one set of vulnerabilities, and reviewers currently have no consistent way to identify which applies to which. The corrective is not a general demand for more disclosure, but the specific disclosure each claim type requires. Table 5 sets out a minimum, non-exhaustive reporting checklist drawn from the preceding five sections. None of it demands new instrumentation; each item is a matter of stating what was already done.

This Perspective has deliberately confined its scope to comparisons made within a single measurement tier, principally rotating-disk and static half-cell characterization. The corrective proposed here does not require new instrumentation, a longer protocol, or a change in technique: it requires stating, for each metric reported, which of the choices catalogued in Table 1 was made. Translating this into practice does not require a new committee or a revised protocol. For authors, it means stating, for each metric reported, which of the choices in Table 5 was made, in the same sentence or table as the value itself, rather than assuming it is implied by citing the original protocol. For reviewers, it means treating a missing entry against Table 5 as an incomplete submission rather than a stylistic omission, and checking that the loading, substrate, and iR-compensation basis behind a headline η_10_ value are stated before treating that value as comparable to a cited literature figure. For editors and journals, it means requiring a short disclosure statement, following the pattern already established for data availability and competing interests, that names the choices in Table 5 rather than leaving them to be inferred from the methods section. None of this requires new instrumentation or a new standards body; each item in Table 5 already exists in the field’s own reporting conventions and needs only to be made explicit alongside the value it qualifies. Realizing it at scale is a community-standards question this Perspective does not resolve on its own: whether through a required checklist at submission, an interlaboratory reference material with an agreed uncertainty bound, or an open, versioned dataset of primary-source values that supersedes citation-chain reuse of unverified numbers is a choice for the field, not for one paper to make. What this Perspective offers is the map of where that choice matters most. Commercial Pt/C, a nominally fixed reference material, spreads by 60 mV across five matched-basis reports. That spread is comparable to the processing and normalization artefacts catalogued in Section 3 and Section 4, and to the margins by which new HER catalysts are often reported to approach or surpass Pt/C. A reference value that moves this much between laboratories cannot, on its own, anchor a cross-laboratory ranking. Closing that gap is a precondition for using overpotential, or any of the other four metrics, to rank catalysts with confidence.

## Figures and Tables

**Table 1:** Six-stage measurement pipeline for HER/OER overpotential comparisons, mapped against the claim type each stage most threatens. Bracketed numbers refer to the main-text reference list.

Stage	Artifact	Magnitude	Claim Type Most Exposed
1. Synthesis/procedure	Interlaboratory reproducibility	~2 orders of magnitude across nominally identical procedures [4] (single interlaboratory study)	Cross-literature record claims
2. Test conditions	pH/temperature ambiguity; interfacial pH	7–30 mV at low j; local pH approaching 1 near 1 A cm^−2^ [5,6,7]	High current density/industrial relevance claims
3. Data processing	iR compensation; Tafel method	13 to >150 mV (10→100 mA cm^−2^); 47 mV manual vs. automated compensation [8]; further sources in Section 4.2 and Section 4.3	Same-lab ranking claims
4. Normalization	TOF basis; site counting; loading normalization	2–3 orders of magnitude (TOF); 26% MPE between the two most concordant site-counting methods [8,10]; 40–60 mV per decade of loading (η_10_)	Mechanistic/active-site claims; same-lab ranking claims
5. Characterization–activity link	Reconstruction	>10× activity change; ≈50% irreversible crystallite shrinkage [12,13]	Before/after stability claims
6. Measurement platform	Operando artifacts	>100 mV reference drift; ms-scale event vs. probe acquisition mismatch [11] (single multi-group perspective)	Operando/working-condition mechanistic claims

Comparisons that advance more than one claim type (for example, a ranking claim and a mechanistic claim in the same study) inherit the vulnerabilities of every stage that applies, not only the one listed as most exposed.

**Table 2:** Substrate, loading, electrolyte, and Tafel slope across seven representative NiFe OER reports. NF, nickel foam; LDH, layered double hydroxide.

Catalyst/Source	Substrate	Loading	Electrolyte	η_10_ (mV)	Tafel (mV dec^−1^)
NiFe/NF, Chen et al.	Ni foam (3D)	not disclosed	1 M KOH	134 ^a^	38.2
Ni_0.5_Fe_0.5_ clusters, Geerts et al.	Gas-phase model electrode	~1 monolayer	1 M KOH	372	37
NiFe LDH, Dionigi et al.	Glassy carbon (planar)	0.1 mg cm^−2^	0.1 M KOH	348	not reported
NiFe, Wang et al.	Stainless steel (3D)	not disclosed	0.1 M KOH	514	not reported
NiFe (40% Fe), Louie and Bell	Au (planar, thin film)	~25–70 nm film	0.1 M KOH	280	~40 ^b^
NiFe_0.5_(OH)_x_, Tang et al.	not specified	not disclosed	1 M KOH	265	not reported
Undoped NiFe LDH, Li et al.	Ni foam (3D)	not given as mass per area	1 M KOH	175	80.32

^a^ Chen et al.’s own headline number is 182 mV at 50 mA cm^−2^; 134 mV is their 10 mA cm^−2^ figure, given in the results text rather than the abstract. ^b^ Reported across mixed Ni-Fe compositions up to 95% Fe, not stated specifically at 40% Fe.

**Table 3:** Site-counting disagreement for the same iron single-atom Fe-N-C catalyst set: three established methods compared against the hydroquinone oxidation reference (four methods in total).

Method Compared to Hydroquinone Oxidation	Mean Absolute Error (10^−5^ mol g^−1^)	Mean Percentage Error
CO pulse chemisorption	1.4	26%
Electrochemical NO stripping	2.3	53%
^57^Fe Mössbauer spectroscopy	comparable, with systematic outliers when FeN^x^ sites are a minority species	—

Data as reported [10]. All four methods were applied to catalysts made by different synthetic routes; none is treated here as ground truth.

**Table 4:** Summary statistics for the two case-study arms (η_10_, 1 M KOH, room temperature basis only).

Arm	n	Mean (mV)	Median (mV)	SD (mV)	CV (%)
OER (NiFe (oxy)hydroxide/LDH)	5	237	237	92	39
HER (commercial Pt/C)	5	59	48	27	46

n, number of sources meeting the matched basis (η_10_, 1 M KOH, room temperature); SD, sample standard deviation (n − 1 denominator); CV, coefficient of variation (SD/mean × 100). Nonparametric bootstrap 95% intervals for the CV: OER 14–52%, HER 11–55%; the two arms cannot be distinguished at this sample size. OER sources: Chen et al. [47], Geerts et al. [48], Li et al. [49], Tang et al. [50], and Jeon et al. [51] (237 mV, results section); using Jeon et al.’s abstract value of 245 mV instead gives a mean of 238 mV, median 245 mV, SD 92 mV, and CV 38%. HER sources: [52,53,54,55,56].

**Table 5:** Minimum reporting checklist by metric.

Metric (Section)	Minimum Disclosure	Why It Matters
Overpotential, η_10_ (§3)	Catalyst loading and substrate geometry alongside any η_10_ value	Geometric normalization without loading/substrate is not comparable across labs
Tafel slope (§4)	Whether extraction was steady-state or potentiodynamic; scan rate if potentiodynamic; overpotential window used for the linear fit	Potentiodynamic extraction underestimates the steady-state slope by about 10–15 mV dec^−1^ under typical screening conditions, enough to blur neighboring mechanistic assignments
ECSA (§5)	Assumed specific capacitance (C_s_) and an explicit ±1-order-of-magnitude uncertainty range, not a single exact value	The method carries at least ±1 order of magnitude uncertainty under its own originators’ accounting
Turnover frequency (§6)	Which of the three common site-counting methods was used; whether the catalyst is known to reconstruct operando, in which case TOF describes the reconstructed rather than the nominal precatalyst	Site-counting choice alone shifts TOF by two to three orders of magnitude
Faradaic efficiency (§7)	Measurement method (GC, water displacement, or RRDE); number of independent measurements averaged; for RRDE, the experimentally calibrated rather than theoretical collection efficiency	The three methods carry materially different, undisclosed error profiles

## Data Availability

The literature values compiled and independently checked for the case study in Section 8 are reported in full in the text and in Tables S1 and S2 (Supporting Information) and summarized in Table 4; Figure 2 and Figure 3 are simulated illustrations computed from Equations (2), (4) and (5) with illustrative parameters; no new experimental data were generated.

## Supplementary Materials

The following supporting information can be downloaded at: https://www.mdpi.com/article/10.3390/molecules31203696/s1, Table S1: OER arm: sources cited in Section 8.1 (undoped or minimally modified NiFe (oxy)hydroxide or layered double hydroxide (LDH) catalysts, alkaline). NF, nickel foam; Table S2: HER arm: sources cited in Section 8.2 and Section 8.3 (commercial Pt/C, alkaline). RT, room temperature; RDE, rotating disk electrode.
