# Supplementary material for: What the Overpotential Does Not Tell You: Diagnosing Non-Comparability Across Hydrogen and Oxygen Evolution Benchmarking Metrics
Source: Molecules. 2026 Oct 15;31(20):3696. doi: 10.3390/molecules31203696 (PMC13707403; doi:10.3390/molecules31203696)
Supplement: Supplementary file 1 [file molecules-31-03696-s001.zip › molecules-4614850-supplementary.pdf]

Supporting Information

What the Overpotential Does Not Tell You: Diagnosing Non-Comparability Across Hydrogen and Oxygen Evolution Benchmarking Metrics

Raymond Tichaona Taziwa* and Jude Nodebechukwunso Ike
Department of Applied Sciences, Faculty of Natural Sciences, Walter Sisulu University, Buffalo City Campus, East London 5200, South Africa
*Corresponding author. E-mail: rtaziwa@wsu.ac.za

This Supporting Information provides the complete literature dataset underlying the case study in Section 8 (“Applying the Framework: A Worked Case Study”) of the accompanying manuscript: Table S1 for the OER arm (undoped/minimally modified NiFe (oxy)hydroxide or LDH catalysts, alkaline) and Table S2 for the HER arm (commercial Pt/C, alkaline). Reference numbers in both tables correspond to the main-text reference list; full citations are reproduced below for convenience.

Table S1. OER arm: sources cited in Section 8.1 (undoped or minimally modified NiFe (oxy)hydroxide or layered double hydroxide (LDH) catalysts, alkaline). NF, nickel foam.

Ref	Catalyst / Source	Substrate	Loading	Electrolyte	η (mV)	Tafel (mV dec^{-1})	Note
[47]	NiFe/NF, Chen et al.	Ni foam (3D)	not disclosed	1 M KOH	η10 = 134a	38.2	a Abstract headline is 182 mV @ 50 mA cm^{-2}; 134 mV is the η10 figure, given only in results text
[48]	Ni0.5Fe0.5 clusters, Geerts et al.	Gas-phase model electrode	~1 monolayer	1 M KOH	η10 = 372	37	Most methodologically distinct entry (gas-phase cluster deposition)
[44]	NiFe LDH, Dionigi et al.	Glassy carbon (planar)	0.1 mg cm^{-2}	0.1 M KOH	η10 = 348	not reported	Flagged: non-standard electrolyte concentration
[45]	NiFe, Wang et al.	Stainless steel (3D)	not disclosed	0.1 M KOH	η10 = 514	not reported	Flagged: non-standard electrolyte concentration
[39]	NiFe (40% Fe), Louie and Bell	Au (planar, thin film)	~25–70 nm film	0.1 M KOH	η10 = 280	~40	Flagged: non-standard electrolyte concentration
[40]	NiFe LDH/NF, Dai et al.	Ni foam (3D)	not disclosed	1 M KOH / 6 M KOH,60°C / artificial seawater / natural seawater	η100 = 233 / 180 / 243 / 280	not reported	Internal control: same catalyst, four electrolyte conditions, no change to catalyst
[50]	NiFe0.5(OH)x, Tang et al.	not stated	not disclosed	1 M KOH	η10 = 265	not reported	Internal control: same paper reports Ni(OH)x 389 mV, Fe(OH)x 430 mV, IrO2 272 mV
[41]	NiFe-LDH/NF, Wang et al.	Ni foam (3D)	not disclosed	1 M KOH	η50 = 239 / η100 = 260	64.1	Avoids η10; authors cite Ni2+/Ni3+ oxidation peak distorting low-j LSV
[42]	NiFe-LDH/NF, Sun et al.	Ni foam (3D)	not disclosed	1 M KOH	η50 = 225 / η100 = 239	41.9	Avoids η10; same distortion issue cited

Ref	Catalyst / Source	Substrate	Loading	Electrolyte	η (mV)	Tafel (mV dec^{-1})	Note
[51]	NiFe/NiO, Jeon et al.	not stated	not disclosed	1 M KOH	η10 = 245 (abstract) / 237 (best, 2nd-stage)	not reported	Two candidate η10 values within one paper; neither flagged as superseding the other; Table 4 uses 237 mV (results section)
[49]	NiFe-LDH-4, Li et al.	Ni foam (hydrothermal)	not given as mg cm^{-2} (grown directly on foam)	1 M KOH	η10 = 175	80.32	Undoped LDH, Fe:Ni = 4:1

Table S2. HER arm: sources cited in Sections 8.2 and 8.3 (commercial Pt/C, alkaline). RT, room temperature; RDE, rotating disk electrode.

Ref	Source	Loading	Electrolyte / Temp	Substrate	η10 (mV)	Tafel (mV dec^{-1})	Note
[43]	Wan et al.	5–200 μg cm^{-2} (loading series)	1 M KOH, RT	Glassy carbon disk (RDE)	no single value (loading series)	33–66 across loading; 63–211 across fitting window at fixed loading	Methodology anchor: same Pt/C, Tafel slope is an artefact of loading and fitting window, not a fixed property
[56]	Zhang et al.	0.050 mg cm^{-2}	1 M KOH, RT	Glassy carbon disk	39	99	Same paper, second condition below
[56]	Zhang et al.	0.10 mg cm^{-2}	5 M KOH, 60°C	Carbon cloth	32.1	not reported	Loading, electrolyte, temperature, and substrate all change together vs. row above
[55]	Sadeghi et al.	~0.20 mg cm^{-2} (reconstructed from ink recipe)	1 M KOH	Ni foam	83	105	No iR correction stated; system resistance 11.7 Ω reported as uncorrected
[54]	Bekmyrza et al.	~0.40 mg cm^{-2} (reconstructed: 1.0 mg cm^{-2} total ink × 40 wt% Pt)	1 M KOH, RT	Ni foam	48	30	Comparator prepared “following the same procedure” as the paper's own catalyst
[52]	Kweon et al.	not disclosed	1 M KOH	not stated	33	43	Ru on multi-walled carbon nanotube (Ru@MWCNT) paper's Pt/C comparator; low end of arm's spread
[46]	Cui and Chen (US Patent 11,855,294 B2)	not disclosed	~30 wt% KOH (~5.3 M)	not stated	71	not reported	Flagged: non-standard, concentrated electrolyte; no 1 M KOH figure given
[53]	Samanta et al.	not disclosed	1 M KOH	not stated	93.4	82.6	High end of η10 range cited in para on Pt/C spread

References cited in Tables S1 and S2 (numbering matches the main manuscript)

39. Louie, M.W.; Bell, A.T. An Investigation of Thin-Film Ni–Fe Oxide Catalysts for the Electrochemical Evolution of Oxygen. *J. Am. Chem. Soc.* **2013**, *135*, 12329–12337. https://doi.org/10.1021/ja405351s.

40. Dai, J.; Zhang, Y.; Song, H.; Liu, L. NiFe Layered-Double-Hydroxide Nanosheet Arrays Grown In Situ on Ni Foam for Efficient Oxygen Evolution Reaction. *Int. J. Hydrogen Energy* **2024**, *87*, 130–137. https://doi.org/10.1016/j.ijhydene.2024.09.042.

41. Wang, L.; Liu, D.; Zhang, Z.; Li, Y.; Liu, J.; Yang, Y.; Xue, B.; Li, F. Self-Adaptively Electrochemical Reconstruction of NiFe-Layered Double Hydroxide on Ni Foam for High-Performance Water Splitting. *J. Alloys Compd.* **2023**, *934*, 167846. https://doi.org/10.1016/j.jallcom.2022.167846.

42. Sun, S.; He, Y.; Chen, T.; Sun, C.; Wu, C. Morphology Regulated Synthesis of NiFe-Layered Double Hydroxide Nanostructures on Nickel Foam toward Efficient Oxygen Evolution Reaction. *J. Alloys Compd.* **2023**, *963*, 171304. https://doi.org/10.1016/j.jallcom.2023.171304.

43. Wan, C.; Ling, Y.; Wang, S.; Pu, H.; Huang, Y.; Duan, X. Unraveling and Resolving the Inconsistencies in Tafel Analysis for Hydrogen Evolution Reactions. *ACS Cent. Sci.* **2024**, *10*, 658–665. https://doi.org/10.1021/acscentsci.3c01439.

44. Dionigi, F.; Zeng, Z.; Sinev, I.; Merzdorf, T.; Deshpande, S.; Lopez, M.B.; Kunze, S.; Zegkinoglou, I.; Sarodnik, H.; Fan, D.; Bergmann, A.; Drnec, J.; de Araujo, J.F.; Gliech, M.; Teschner, D.; Zhu, J.; Li, W.-X.; Greeley, J.; Roldan Cuenya, B.; Strasser, P. In-Situ Structure and Catalytic Mechanism of NiFe and CoFe Layered Double Hydroxides during Oxygen Evolution. *Nat. Commun.* **2020**, *11*, 2522. https://doi.org/10.1038/s41467-020-16237-1.

45. Wang, Y.; Williamson, N.; Dawson, R.; Bimbo, N. Electrodeposition of Nickel–Iron on Stainless Steel as an Efficient Electrocatalyst Coating for the Oxygen Evolution Reaction in Alkaline Conditions. *J. Appl. Electrochem.* **2023**, *53*, 877–892. https://doi.org/10.1007/s10800-022-01817-4.

46. Cui, Y.; Chen, W. Metal-Hydrogen Batteries for Large-Scale Energy Storage. U.S. Patent 11,855,294 B2, 26 December 2023.

47. Chen, Y.; Zeng, X.; Meyer, Q.; Zhao, C.; He, Z.; Wu, F.; Tang, H.; Cheng, Y. An Outstanding NiFe/NF Oxygen Evolution Reaction Boosted by the Hydroxyl Oxides. *Electrochim. Acta* **2023**, *442*, 141862. https://doi.org/10.1016/j.electacta.2023.141862.

48. Geerts, L.; Cosentino, S.; Liao, T.-W.; Yadav, A.; Lin, P.-C.; Zharinov, V.S.; Hu, K.-J.; Longo, A.; Pereira, L.M.C.; Grandjean, D.; Rongé, J.; Lievens, P.; Martens, J.A. Highly Active Oxygen Evolution Reaction Model Electrode Based on Supported Gas-Phase NiFe Clusters. *Catal. Today* **2019**, *334*, 59–67. https://doi.org/10.1016/j.cattod.2019.01.062.

49. Li, X.; Xu, S.; Li, J.; Zhang, S.; Zhang, B.; Zhao, R.; Zhao, D.; Wu, F. NiFe-LDH as a Bifunctional Electrocatalyst for Efficient Water and Seawater Electrolysis: Enhanced Oxygen Evolution and Hydrogen Evolution Reactions. *Nanoscale Adv.* **2025**, *7*, 5546–5560. https://doi.org/10.1039/D5NA00350D.

50. Tang, T.; Jiao, S.; Han, J.; Wang, Z.; Guan, J. Partially Crystallized Ni–Fe Oxyhydroxides Promotes Oxygen Evolution. *Int. J. Hydrogen Energy* **2023**, *48*, 5774–5782. https://doi.org/10.1016/j.ijhydene.2022.11.118.

51. Jeon, J.-H.; Kim, J.-E.; Kim, T.-H.; Park, C.-S.; Jung, K.; Yoon, J.; Kim, J.; Kim, Y.-H.; Kang, K.-S. Enhanced Oxygen Evolution Reaction in Hierarchical NiFe/NiO Electrocatalysts: Effects of Electrodeposition Condition on Electrode. *Electrochem. Commun.* **2024**, *160*, 107668. https://doi.org/10.1016/j.elecom.2024.107668.

52. Kweon, D.H.; Okyay, M.S.; Kim, S.-J.; Jeon, J.-P.; Noh, H.-J.; Park, N.; Mahmood, J.; Baek, J.-B. Ruthenium Anchored on Carbon Nanotube Electrocatalyst for Hydrogen Production with Enhanced Faradaic Efficiency. *Nat. Commun.* **2020**, *11*, 1278. https://doi.org/10.1038/s41467-020-15069-3.

53. Samanta, R.; Manna, B.K.; Trivedi, R.; Chakraborty, B.; Barman, S. Hydrogen Spillover Enhances Alkaline Hydrogen Electrocatalysis on Interface-Rich Metallic Pt-Supported MoO_3. *Chem. Sci.* **2024**, *15*, 364–378. https://doi.org/10.1039/D3SC04126C.

54. Bekmyrza, K.Z.; Kuterbekov, K.A.; Kabyshev, A.M.; Kubenova, M.M.; Baratova, A.A.; Aidarbekov, N.; Chaka, M.D.; Benti, N.E. High-Performance Hydrogen Energy Generation via Innovative Metal–Organic Framework Catalysts and Integrated System Design. *Sci. Rep.* **2025**, *15*, 28418. https://doi.org/10.1038/s41598-025-08306-6.

55. Sadeghi, E.; Chamani, S.; Yildirim, I.D.; Erdem, E.; Peighambardoust, N.S.; Aydemir, U. In Situ Design of a Nanostructured Interface between NiMo and CuO Derived from Metal–Organic Framework for Enhanced Hydrogen Evolution in Alkaline Solutions. *ACS Appl. Mater. Interfaces* **2024**, *16*, 10078–10092. https://doi.org/10.1021/acsami.3c17588.

56. Zhang, R.; Li, Y.; Zhou, X.; Yu, A.; Huang, Q.; Xu, T.; Zhu, L.; Peng, P.; Song, S.; Echegoyen, L.; Li, F.-F. Single-Atomic Platinum on Fullerene C_{60} Surfaces for Accelerated Alkaline Hydrogen Evolution. *Nat. Commun.* **2023**, *14*, 2460. https://doi.org/10.1038/s41467-023-38126-z.
